# Violet LED light enhances the recruitment of a thrip predator in open fields

**DOI:** 10.1038/srep32302

**Published:** 2016-09-08

**Authors:** Takumi Ogino, Takuya Uehara, Masahiko Muraji, Terumi Yamaguchi, Takahisa Ichihashi, Takahiro Suzuki, Yooichi Kainoh, Masami Shimoda

**Affiliations:** 1 Institute of Agrobiological Sciences, NARO; Ohwashi 1–2, Tsukuba, Ibaraki 305-8634, Japan; 2 Graduate School of Life and Environmental Sciences, University of Tsukuba, Tennodai 1-1-1, Tsukuba, Ibaraki 305-8572, Japan; 3 SHIGRAY Inc., Sumida, Tokyo, Japan

## Abstract

The predatory bug *Orius sauteri* is an indigenous natural enemy of thrips and whiteflies in Asian countries. To put these bugs to practical use in pest management, methods to attract and retain the bugs in agricultural fields are needed. We previously showed that violet light (405 nm) attracts *O. sauteri* selectively. Many thrips and whiteflies are attracted to UV or green light. In this study, we examined the effect of violet-LED illumination on *O. sauteri* in pesticide-free eggplant (*Solanum melongena* L.) cultivation. In three cultivation trials, the density of *O. sauteri* on eggplant leaves was consistently higher in the illuminated plots; at least twice that of the non-illuminated plots. Simultaneously, the density of thrips declined markedly to less than half that of the non-illuminated plots. We identified three positive effects of violet light including an “immediate-effect” on predator attraction, a “persistent-effect” on predator reproduction, and a “secondary-effect” on the food web structure. Our results showed that illumination with violet light provides a powerful tool for integrated pest management. This is the first report on the use of illumination to manipulate the behavior of natural enemies.

Insecticide resistance has become an enormous threat to agricultural production. Both thrips and aphids have developed resistance to neonicotinoids, a recently developed insecticide group^1^,^2^,^3^. One possible solution for this serious problem is to introduce natural enemies as biological control agents. Therefore, numerous efforts have been made to characterize various natural enemies, such as predatory insects^4^ and parasitoids^5^,^6^, and to control pests by releasing these natural enemies into agricultural fields^7^. Recently, in consideration of the effects on biodiversity, the use of indigenous natural enemies as part of conservation biological control (CBC) has been proposed^8^,^9^, and has become established by altering cultivation methods and landscapes.

The minute pirate bug (*Orius* species, Heteroptera: Anthocoridae) is a tiny predatory insect against thrips and whiteflies, and is distributed worldwide^10^,^11^,^12^,^13^,^14^. This species is an effective and beneficial natural enemy in pest management^4^,^15^: For example, *O. insidiosus* (Say) and *O. tristicolor* (White) are commercially available as biological control agents in North America^16^ and *O. laevidatus* is available in Europe^17^. In Asian countries, *O. strigicollis* is distributed in warm regions of Japan^11^,^18^ and has been commercially supplied since 2001^19^.

Usage of these biological control agents is limited to greenhouse farming. In contrast, *O. sauteri* is the most common species throughout Japan^19^ and preys on agricultural micro pests^20^,^21^. Therefore, *O. sauteri* is expected to be effective for pest control not only in the greenhouse, but also in outdoor cultivation^22^,^23^.

In the past decade, there have been various attempts to attract *O. sauteri* to cultivated fields. Some plants, such as rudbeckia, were evaluated as a banker plant for this insect^24^,^25^,^26^. Another potential tool for attraction is sex pheromones; however, none have been identified^27^. Recently, applications of light or color control have been identified as pest management methods^28^,^29^. In a previous study, we investigated the phototaxis behavior of *O. sauteri*, and showed that the bug is strongly attracted to violet light (405 nm)^30^. In general, many insects are attracted to light containing ultraviolet (UV, <380 nm)^31^,^32^ or green (525 nm)^33^,^34^, consistent with peaks in the light sensitivity of the compound eyes of insects^35^. Therefore, violet light is a promising candidate for selectively attracting natural enemies to an agricultural field. In this study, we performed pesticide-free eggplant cultivation using violet-LED illumination and evaluated the effectiveness of violet illumination as a new tool for pest management.

## Results

**Figure 1:**
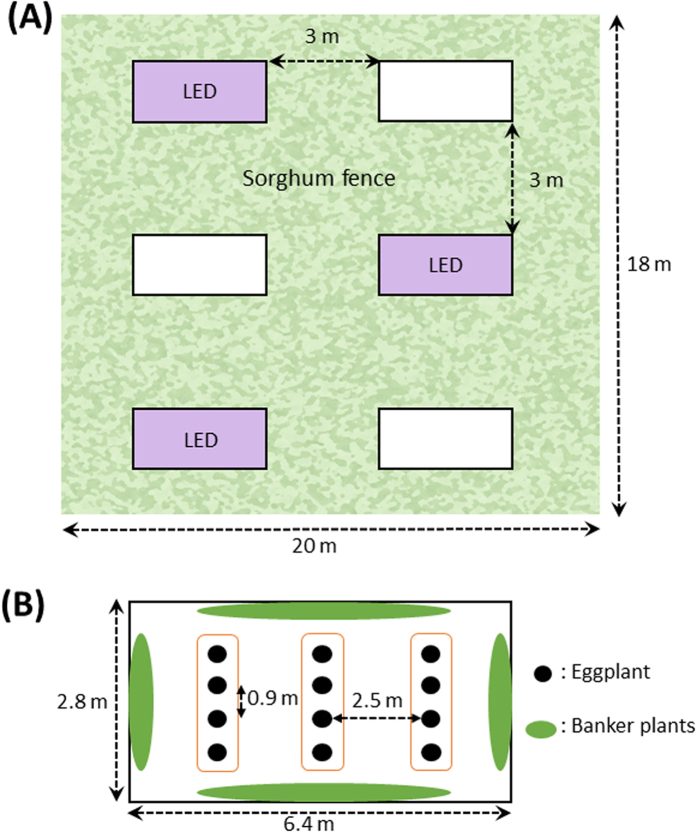
Plot design and locations of experimental plots in the eggplant field. (**A**) The experimental field had six plots which included three illuminated plots and three non-illuminated plots. Sorghum fence approximately 3 m in width were planted to separate each experimental plot to minimize inter-plot interference. (**B**) There were three rows of eggplant per plot. Four eggplant trees were planted in a row. Banker plants were planted surrounding eggplant rows. Space between eggplants was 0.9 m in the row, and one plot consisted of three rows 2.5 m apart.

We divided an eggplant field into two plots; violet illuminated and non-illuminated (Fig. 1), then observed the occurrence of *O. sauteri* and thrips in these plots. A rope-type LED light source (3 m long) was used. *O. sauteri* and thrips on ten eggplant leaves were captured using square sticky paper. The numbers of catches in both plots were compared to verify whether *O. sauteri* was attracted to illuminated plants and whether the population density of thrips was suppressed. We conducted three trials: In Trial 1, three ropes of LEDs were lit, from June 29 to July 30. In Trial 2, three ropes of LEDs were lit from August 4 to October 9. In Trial 3, one rope of LEDs was lit from August 19 to October 10. In all trials and throughout the experimental period, *O. sauteri* and thrips species that were captured were identified to be *O. sauteri* and *Mycterothrips glycines* (Okamoto), respectively, based on their morphologies. Mean temperatures and precipitation in Tsukuba City during the experimental period were measured at the Aerological Observatory which is located 2.8 km from the experimental field with an elevation difference of 3.2 m.

### Field Trial 1

**Figure 2:**
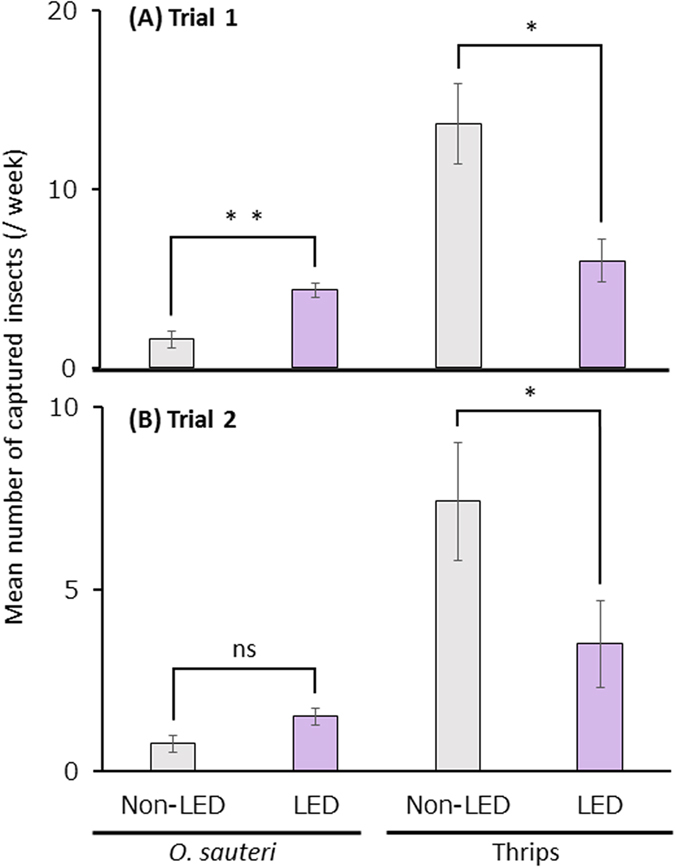
The densities of *Orius sauteri* and thrips in plots with and without LED. Mean numbers of *O. sauteri* and thrips captured per week in Trial 1 (**A**) and Trial 2 (**B**). Vertical bars indicate standard error (A, n = 12; B, n = 12). Statistical analysis was by the Mann-Whitney U-test. * and ** indicate statistical significance at *p* < 0.05 and *p* < 0.01, respectively.

During the experimental period, the total numbers of *O. sauteri* and thrips captured in the illuminated plot were 57 and 74, respectively, whereas those captured in the non-illuminated plot were 20 and 183, respectively. In the illuminated plot, the numbers of *O. sauteri* were 2.9 times greater, and the numbers of thrips were reduced by 60% of the value for the non-illuminated plot. The numbers of *O. sauteri* and thrips captured per week in the illuminated plots were 4.33 ± 0.43 and 6.00 ± 1.21 (mean ± SE, n = 12), respectively (Fig. 2A). Those captured in the non-illuminated plots were 1.58 ± 0.49 and 13.67 ± 2.23, respectively (Fig. 2A). From these results, a greater number of *O. sauteri (p* < 0.01) and smaller number of thrips (*p* < 0.05) were always captured in the illuminated plot.

**Figure 3:**
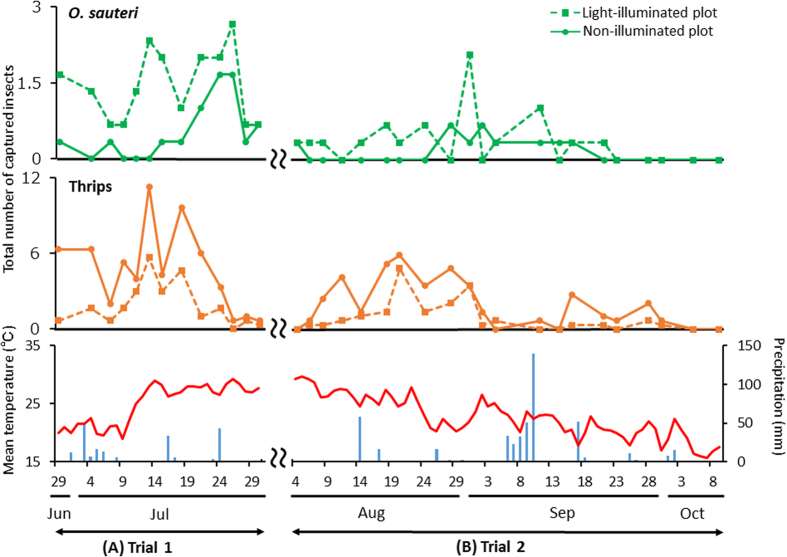
Population trends for *Orius sauteri* and thrips. Total number of *O. sauteri* (upper panel) and thrips (middle panel) captured in Trial 1 (**A**) and Trial 2 (**B**). The mean temperature and precipitation in Tsukuba City during the experimental period (lower panel).

Next, we investigated temporal variation in the number of captured *O. sauteri* and thrips (Fig. 3A). After three days from the start of lighting, the numbers of *O. sauteri* captured in the illuminated and non-illuminated plots were 5 and 1, respectively. The numbers of thrips captured in the illuminated and non-illuminated plots were 2 and 19, respectively. The lower density of *O. sauteri* corresponded to an increased number of thrips, and gradually increased after July 9 in both plots. In the illuminated plots, the density of *O. sauteri* was at least twice that of the non-illuminated plots until the end of Trial 1. The density of thrips in the illuminated plots was always less than 75% of that in the non-illuminated plots. The densities of *O. sauteri* and thrips decreased temporarily on July 7 because of a short period of hard rain during the previous night (Fig. 3A, lower panel). After July 28, the densities of *O. sauteri* and thrips were reduced drastically, due to increased temperature and drought.

According to Matsuzaki and Ichikawa^36^, the density of 0.73 thrips/leaf (assumed to be 400 cm^2^) causes a 10% loss of eggplant yield, which is usually considered the economic threshold in Japan. In this study, 1.82 thrips/leaf and 0.8 thrips/leaf were captured in non-illuminated and illuminated plots, respectively, in July. Although thrip density was reduced significantly in illuminated plots, the densities of thrips in both plots were clearly higher than the economic threshold.

### Field Trial 2

After Trial 1, the eggplants grew into large trees. Branches were pruned back, according to typical practice for eggplant cultivation in Japan. The total numbers of *O. sauteri* and thrips captured in the illuminated plots were 21 and 53, respectively, whereas in the non-illuminated plots they were 10 and 118, respectively. In the illuminated plots, there were 2.1 times more *O. sauteri* than in the non-illuminated plots. There were more thrips in the non-illuminated plots than the illuminated plots, and there were half the number of thrips in the illuminated plots compared to the non-illuminated plots. The numbers of *O. sauteri* and thrips captured per week in the illuminated plots were 1.50 ± 0.22 and 3.50 ± 1.19 (mean ± SE, n = 12), respectively, and 0.75 ± 0.24 and 7.42 ± 1.62, respectively, in the non-illuminated plots (Fig. 2B). From these results, a larger number of *O. sauteri* (N.S.) and a smaller number of thrips (*p* < 0.05) were captured in the illuminated plots in trial 2.

Next, we investigated temporal variation in the numbers of captured *O. sauteri* and thrips (Fig. 3B). From August 4 to 13, insects were rarely found in both plots because of dry weather and high temperatures. During the first day of the investigation, the number of *O. sauteri* captured in both plots was 1. No *O. sauteri* were captured in the non-illuminated plots from August 6 to August 24. In the illuminated plots, no *O. sauteri* were captured until August 11; however, the density recovered on August 14. In contrast, thrips were observed in both plots after August 6. In the illuminated plots, the density of thrips was maintained at a lower level than in the non-illuminated plots until September 30. *O. sauteri* could not be captured, since the mean temperature was lower than 20 °C (Fig. 3B, lower panel).

As for the economic threshold of thrips density, 1.12 and 0.60 thrips/leaf were captured in non-illuminated and illuminated plots, respectively, in August. In September, 0.40 and 0.12 thrips/leaf were captured in non-illuminated and illuminated plots, respectively. In Trial 2, the thrip density in non-illuminated plots was higher than the economic threshold in August.

### Field Trial 3

**Figure 4:**
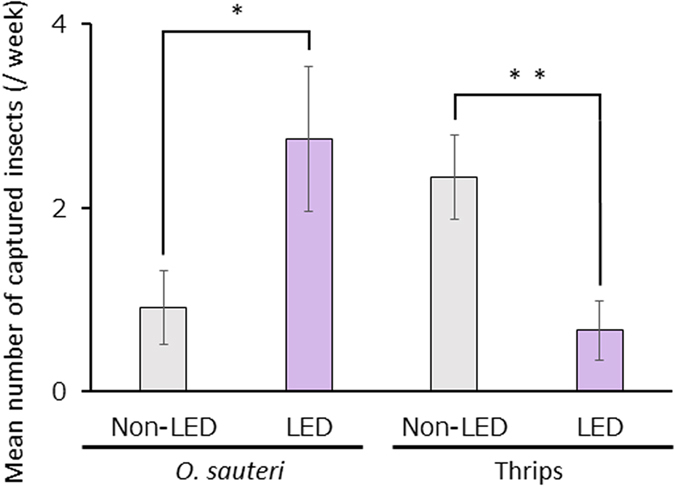
The densities of *Orius sauteri* and thrips in plots with and without LED. Mean numbers of *O. sauteri* and thrips captured per week after turning on the LED light source in Trial 3. Vertical bars indicate standard error (n = 12). Statistical analysis was by the Mann-Whitney U-test. * and ** indicate statistical significance at *p* < 0.05 and *p* < 0.01, respectively.

In Trial 3, we reduced the number of rope LED lights by two-thirds, to examine the effects of violet light. During the experimental period, the total numbers of *O. sauteri* and thrips captured in the illuminated plots were 47 and 16, respectively, while those captured in the non-illuminated plot were 21 and 43, respectively. The total numbers of *O. sauteri* captured in the illuminated plots were 2.7 times greater than in the non-illuminated plots. The total numbers of thrips in the illuminated plots were reduced by 70% of the value for the non-illuminated plots. The mean numbers of *O. sauteri* and thrips captured per week after turning on LED light are shown in Fig. 4. The numbers of *O. sauteri* and thrips captured weekly in the illuminated plots were 2.75 ± 0.79 and 0.67 ± 0.32 (mean ± SE, n = 12), respectively, and 0.92 ± 0.40 and 2.33 ± 0.46, respectively, in the non-illuminated plots. From these results, a greater number of *O. sauteri (p* < 0.05) and a smaller number of thrips (*p* < 0.01) were always captured in the illuminated plots. These results showed that the effect of violet was valid even if the number of LED lights was reduced by two-thirds.

**Figure 5:**
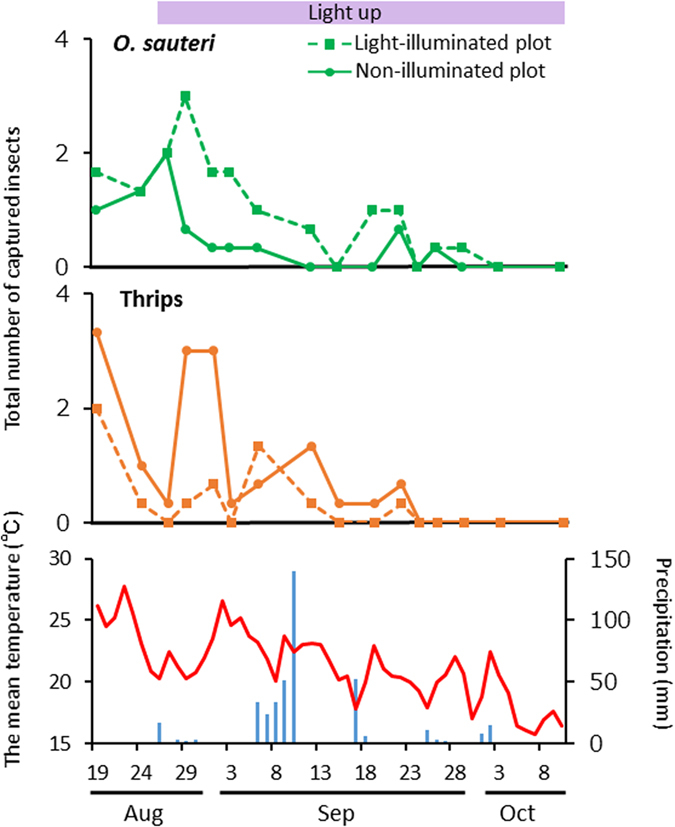
Population trends for *Orius sauteri* and thrips. Total number of *O. sauteri* (upper panel) and thrips (middle panel) captured in Trial 3. The mean temperature and precipitation in Tsukuba City during the experimental period (lower panel). LED light was turned on beginning August 26.

Next, we investigated temporal variation in the numbers of captured *O. sauteri* and thrips (Fig. 5). After three days from the start of lighting, the numbers of *O. sauteri* captured in the illuminated and the non-illuminated plots were 9 and 2, respectively. In contrast, the numbers of thrips captured in the illuminated and non-illuminated plots were 1 and 9, respectively, confirming that the effects of violet illumination had already begun after three days from the start of lighting, as was the case for Trial 1. Except for September 15 and 24, the density of *O. sauteri* in the illuminated plots was higher than in the non-illuminated plots until October 3. As with Trial 2, no *O. sauteri* were captured at that time, since the mean temperature was lower than 20 °C. The changes in the densities of *O. sauteri* and thrips were concurrent with decreases in atmospheric temperature. On September 6, the thrip density in the illuminated plots increased temporally greater than that in the non-illuminated plots (Fig. 5, middle panel). Except for that day, the density of thrips remained lower than in the non-illuminated plots after the first peak, until September 24.

As for the economic threshold of thrip density, 0.27 and 0.11 thrips/leaf were captured in non-illuminated and illuminated plots, respectively, in September. In Trial 3, the thrip densities in both plots remained lower than the economic threshold in September.

**Figure 6:**
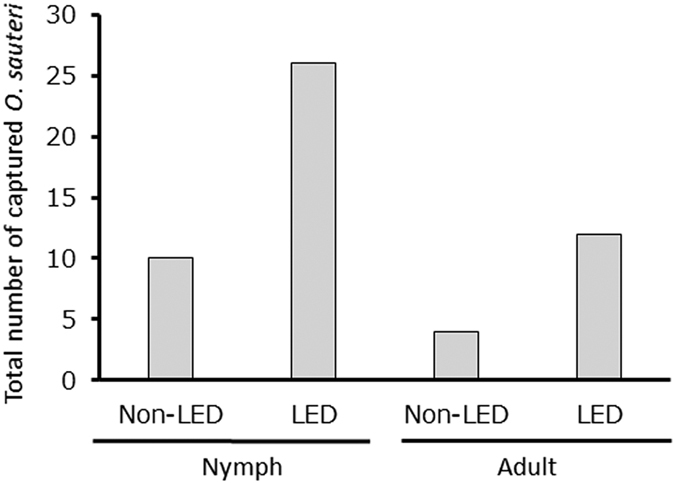
Total numbers of *Orius sauteri* nymphs and adults captured after turning on LED light in Trial 3. Comparison between illuminated plots (LED) and non-illuminated plots (Non-LED).

We further investigated whether the *O. sauteri* captured in Trial 3 were adults or nymphs, to examine the age structure. *O. sauteri* captured in the illuminated plots consisted of 26 nymphs and 21 adults, while those captured in the non-illuminated plots consisted of 11 nymphs and 10 adults (Fig. 6). These results indicated that even a single light illumination can attract and retain a greater number of *O. sauteri*, but it does not affect the age structure.

### Confirmation of predation using genomic PCR

**Figure 7:**
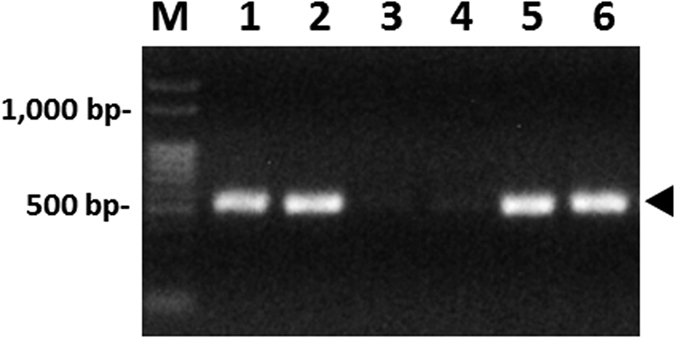
Detection of undigested thrip DNA from the predator body. A 0.5 kb-long, thrip-specific mitochondrial DNA fragment was amplified from *Orius sauteri* whole body extract. M: 100-bp ladder DNA size maker; lanes 1–5: PCR products amplified from *O. sauteri* individuals; lane 6: positive control PCR product amplified from thrips, *M. glycines*. Arrowhead indicates the size of thrip-specific DNA.

To obtain direct evidence for predation of *M. glycines by O. sauteri*, we attempted to amplify undigested prey DNA from the predator by genomic PCR. A 0.5 kb-long thrip-specific mitochondrial DNA fragment, containing the *16S rDNA* and *COX1* genes^37^, was amplified from *O. sauteri* whole-body, total DNA by nested PCR. The PCR products were extracted from electrophoresed gels and sequenced as described in Muraji and Nakahara (2001)^38^. Among 14 *O. sauteri* individuals examined by PCR, using the thrips-specific primer sets, eight individuals (57%) showed DNA bands expected for thrip species (Fig. 7). The nucleotide sequence of the *COX1* gene included in the PCR product was homologous to previously reported sequences of thysanopteran species, such as *Frankliniella occidentalis* (KJ576887) and *Scirtothrips dorsalis* (KM355444), and in agreement with that of the *M. glycines* (DDBJ accession number: LC163947) obtained in this study. These results demonstrate that most *O. sauteri* fed on *M. glycines* in our open field. In the case of flower thrips, *F. intonsa*, the prey DNA was digested quickly within approximately 12–24 hours after predation by *Orius* species (Muraji, unpublished data). Thus, the predation rate was substantially higher than the detection rate of 57%. Our estimate of high predation rate suggests a considerable level of predation pressure on the *M. glycines* population.

## Discussion

Illumination has been applied to pest management based on insect responses to light sources^28^,^35^. Recently LED lights have become popular as a new light source. For example, green LED lights were used effectively as a trap to capture whiteflies [*Bemisia tabaci* (Gennadius)] in tomato plant greenhouses^39^, and sweet potato weevils [*Euscepes postfasciatus* (Fairmaire)]^40^. Most previous studies have used lighting to target and prevent pest species directly^33^,^34^,^41^. In contrast, we tried to control the natural enemy by LED lighting for the suppression of pests. Consequently, we succeeded in increasing the *O. sauteri* density compared to the control. Furthermore, based on the analysis of undigested thrip DNA in the predator, we showed that most *O. sauteri* (57% at least) preyed on the thrips. These results suggested the considerable level of predation pressure on the population of thrips in the eggplant fields. This is the first report of the use of illumination to enhance the recruitment of thrip predators in the open field.

Based on our results using violet-LED illumination, we found that violet lighting provides three positive benefits for pest management. The first confirmed effect was the “immediate-effect” which attracts the *O. sauteri* adults to the eggplants. In the illuminated plots of Trial 1, the density of *O. sauteri* was 5.0 times greater than that in the non-illuminated plots three days following the start of lighting. Although the *O. sauteri* density decreased once in both the illuminated and non-illuminated plots in Trial 2 due to pruning back, the *O. sauteri* density recovered quickly in the illuminated plots. In Trial 3, the density of *O. sauteri* adults in the illuminated plots was 4.5 times higher than that in the non-illuminated plots, just three days after the start of lighting. These results showed clearly that the *O. sauteri* adults moved quickly into the illuminated eggplants from the banker plants. The ability and efficiency of natural enemies to move into crops is important for reducing the time-lag between pest outbreak and control by natural enemies^42^. In a previous study, we observed a rapid response to the light source in laboratory scale experiments^30^. The “immediate-effect” reflects the strong attraction to violet LED light of *O. sauteri* adults and the rapid recruitment of indigenous natural enemies into the crops in our experiments.

The second confirmed effect was the “persistent-effect” which supports the retention of *O. sauteri* adults and their reproduction on the eggplants. The density of *O. sauteri* nymphs in the illuminated plots was 2.6 times greater than that in the non-illuminated plots in Trial 3. This observation indicates that the light attracted adults laid eggs on eggplants and their offspring grew on them. In contrast, the density of thrips was less than half of that in the non-illuminated plots. It was reported previously that early fifth nymphs of *O. sauteri* consumed more thrips than adult *O. sauteri*^43^. Although the age structures of *O. sauteri* in the illuminated and non-illuminated plots were similar, the total numbers of *O. sauteri* nymphs were high in the illuminated plots. Therefore, the decline of thrip density in the latter half of each experiment appears to be have been caused mainly by *O. sauteri* nymphs. The reproduction and generational changes on eggplants are important as “persistent-effects” in using indigenous natural enemies for a long-period of biological control.

In addition to effects on the density of *O. sauteri* and thrips, we found that light illumination had a “secondary-effect” on the food web. In our experimental fields, other herbivorous insects [e.g., leafhoppers (*Chrysopa* spp.) and aphids (*Aphis* spp.)] were observed, but their densities were suppressed to a low level. Who preyed on these pests? We also observed many tree frogs (*Hyla japonica*) on eggplant leaves, from early summer to autumn. This species is well known as a predator in Japan^44^. They are large feeders and move easily from water to dry land, where food resources are abundant^45^. In our observations, this species was the top predator in the experimental fields. This means that frogs suppressed the increase of herbivorous insects, including leafhoppers, directly or indirectly. The number of frogs in the illuminated plots was 2.4 times greater than that in the non-illuminated plots, and the densities of leafhoppers and aphids were the inverse. In previous studies, it was verified that the top predators could predominantly suppress the herbivores and decrease plant damage in the fields^46^,^47^,^48^. Furthermore, the biodiversity derived from the abundant food resources is important in pest control in the cultivation fields of rice, coffee, and cacao^49^,^50^. Although our study is not sufficient to understand overall effects on the food web structure, this “secondary-effect” suggested consequential influences on the agroecosystem.

As a factor interfering with the effects of the violet lighting, weather condition could not be ignored. The *O. sauteri* density was very dependent on the weather. In this study, we established the importance of two meteorological factors. The first factor was rainfall. A drought period after July 28 brought an extreme reduction in *O. sauteri* density in both plots. The other factor was temperature. Lower temperature was concurrent with reduced *Orius* density after September 23. On the other hand, the attractive effect of LED light was observed continuously, except for during the periods mentioned above.

Manipulating the behavior and habitat of natural enemies is essential for success in CBC^51^,^52^. Plant odor treatment in the field is an attempt at such manipulation^53^,^54^,^55^,^56^. Recently, it was reported that the combination of plant odor and flower resources, such as nectar and pollen, could attract and retain natural enemies^57^,^58^. In this study, we showed an alternative method for such manipulation. Violet lighting facilitates both the attraction and retention of indigenous natural enemies on crops. We identified three positive effects of violet lighting, namely the “immediate-effect” of predator attraction, the “persistent-effect” of predator reproduction on crops, and the “secondary-effect” on the food web structure. Although there is ample room for improvement in the practical application of this approach, we have demonstrated the potential of illumination to control natural enemies as a tool for pest management.

## Materials and Methods

### Insects and Plants

Eggplant (*Solanum melongena* L. cv. Senryo-Nigo) was raised in a greenhouse until development into a nursery tree, planted in the experimental field and watered by rainfall. Sesame (*Sesamum indicum*), buckwheat (*Fagopyrum esculentum*), okra (*Abelmoschus esculentus*), marigold (*Tagetes patula*), blue salvia (*Salvia farinacea*), and scaevola (*Scaevola aemula*) served as banker plants^59^. Naturally occurring insects on eggplants were captured, and the species and their numbers were recorded. We did not release any insects; however, banker plants were planted to increase the density of *O. sauteri*. The schedules of manipulation for each trial are shown in Supplementary Table 1.

### Field Trials

All experiments were conducted in an experimental field of the Institute of Agrobiological Sciences, NARO, Tsukuba, Ibaraki, Japan (36.3°N, 140.5°E). The experimental field was divided into six plots (6.4 × 2.8 m) in a 2 × 3 grid, and consisted of three illuminated plots and three non-illuminated plots (Fig. 1A). A two-factor randomized block design was used for all experiments with an ‘illuminated block’ and ‘non-illuminated block’ as factors. There were three replications, with a spacing sorghum (*Sorghum bicolor*) fence approximately 3 meters in width between plots. To prevent influence from neighbor plots, we chose a sorghum variety that grows to a height of more than two meters and was planted as densely as possible. A plot consisted of three rows in which four eggplant trees were planted. Banker plants were planted surrounding eggplant rows. Sesame and buckwheat were seeded at 0.45 m^2^, whereas 12 roots of okra, marigold, and blue salvia were planted. These plants were arranged uniformly on each side of the plot to eliminate positional effects. Three trials, which differed in experimental periods, illumination conditions, and species of banker plants, were performed:

#### Trial 1

A field trial was conducted from June 29 to July 30, 2015. Sesame, okra, buckwheat, marigold, and blue salvia were planted as banker plants in all plots. In the illuminated plots, three LED ropes were used. Accordingly, all rows in each illuminated plot were illuminated. The numbers of *O. sauteri* and thrips on eggplant leaves were counted and compared with those in non-illuminated plots.

#### Trial 2

Except for the experimental period and eggplant condition, this experiment was identical to Trial 1. This trial was conducted from August 4 to October 9, 2015. All eggplant trees were pruned back before starting the experiments according to common practice in this plot of Japan for autumn harvesting.

#### Trial 3

This trial was performed at a different experimental field than the other trials and was conducted in essentially the same manner as trials 1 and 2. The experimental period was from August 19 to October 10, 2015. In addition to the banker plants used in trials 1 and 2, two roots of the scaevola were also planted. To evaluate the efficiency of LED light, the number of LEDs was reduced to one-third that of trials 1 and 2. In addition, the numbers of *O. sauteri* adults and nymphs were counted to determine the age structure in the illuminated plots.

### Illumination setup

**Figure 8:**
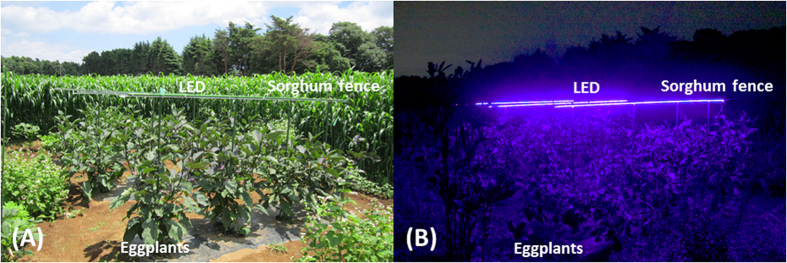
Photographs of experimental plots. (**A**) Daytime view of eggplant field; (**B**) Nighttime view of eggplant field with LED illumination.

A custom-ordered rope light source (3 m long, SHIGRAY Inc., Tokyo, Japan), equipped with LED lights with a peak at 405 nm, was used to illuminate eggplants. The LED rope was mounted on supporting poles above the eggplants (1.4 m from the ground) to illuminate the entire plants. The photon flux density was 0.821 μmol m^−2^ s^−1^ at 30 cm from the light source. Lighting time was from 17:00 to 20:00, when *O. sauteri* activity was high (Fig. 8).

### Method of capture

Insect species and densities on eggplant leaves were investigated using a piece of sticky paper (10 × 10 cm). This method allowed us to investigate an insect density on a unit area (100 cm^2^) and eliminate observation errors. The leaf was chosen randomly and sandwiched in the folded sticky paper. The paper was softly pressed with a finger to capture insects on the glue surface of sticky paper. For a plot, 10 pieces of the paper were used. Eggplant was observed every two or three days to minimize the impact on insect density. The number of insects per species was recorded. All investigations were performed from 9:00 to 12:00 a.m. when *O. sauteri* activity is very low, to prevent insect escape. The numbers of *O. sauteri* and thrips were compared between illuminated and non-illuminated plots, to evaluate the effectiveness of a violet-light illumination.

### Statistical analysis

The data (2 or 3 investigations/week) for each plot were pooled, then weekly counts were analyzed to eliminate daily fluctuation in data. Statistical significance of differences in the numbers of captured insects between illuminated and non-illuminated plots was tested using Mann-Whitney’s U test (Wilcoxon’s rank sum test; one-tailed). Statistical analyses were performed with R 3.2.3 (R Core Team, 2015).

### Confirmation of predation by genomic PCR

Predation of *M. glycines* by *O. sauteri* was confirmed by genomic PCR used to amplify undigested prey DNA from the predator. A 0.5 kb-long, thrips-specific mitochondrial DNA fragment, containing the *16S rDNA* and *COX1* genes^37^, was amplified from *O. sauteri* whole-body, total DNA using nested PCR. Primers used in the first (5′-ACATGATTTGAGTTAAGACCG-3′ and 5′-CATTATAGCGTAAATTATTCCT-3′) and the second PCR (5′-GTGAGCCAGGTTGGTTTCTATC-3′ and 5′-AACTGTTCATCCTGTTCCTGC-3′) were designed based on the similarity among the aligned homologous sequences of *Thrips imaginis Bagnall* (AF335993), *Frankliniella occidentalis* (Pergande) (JN835456), *Frankniella intonsa* (Trybom) (JQ917403), and several other thysanopteran insects. Template DNA was extracted from the entire insect body, dried on a sticky paper trap, using a DNeasy Blood & Tissue Kit (QIAGEN Inc., Valencia, California, USA), and dissolved in 100 μL of sterilized distilled water. PCR was performed using Tks Gflex DNA polymerase (Takara Bio, Shiga, Japan), according to the manufacturer’s instructions. The first PCR product was diluted 20 times, using sterilized Milli-Q water, and as a template for the second PCR. Products of the second PCR were extracted from electrophoresed gels and sequenced using a primer 5′-AACTGTTCATCCTGTTCCTGC-3′, as described in Muraji and Nakahara^38^. To compare nucleotide sequences between the predator and the prey, PCR products of several *M. glycines* individuals were also sequenced.

## Additional Information

**How to cite this article**: Ogino, T. *et al.* Violet LED light enhances the recruitment of a thrip predator in open fields. *Sci. Rep.* **6**, 32302; doi: 10.1038/srep32302 (2016).

## Supplementary Material

Supplementary Table 1

Supplementary Table 2

Supplementary Figure 1
