# Author Correction: Violet LED light enhances the recruitment of a thrip predator in open fields

**DOI:** 10.1038/s41598-020-78514-9

**Published:** 2020-12-08

**Authors:** Takumi Ogino, Takuya Uehara, Masahiko Muraji, Terumi Yamaguchi, Takahisa Ichihashi, Takahiro Suzuki, Yooichi Kainoh, Masami Shimoda

**Affiliations:** 1 Institute of Agrobiological Sciences, NARO, Ohwashi 1–2, Tsukuba, Ibaraki 305-8634 Japan; 2 Graduate School of Life and Environmental Sciences, University of Tsukuba, Tennodai 1-1-1, Tsukuba, Ibaraki 305-8572 Japan; 3 SHIGRAY Inc., Sumida, Tokyo, Japan

Correction to: *Scientific Reports* 10.1038/srep32302, published online 08 September 2016

This Article contains an error in the description of the scales of the experimental filed in Figure 1. The correct Figure 1 appears below as Figure 1.

**Figure 1:**
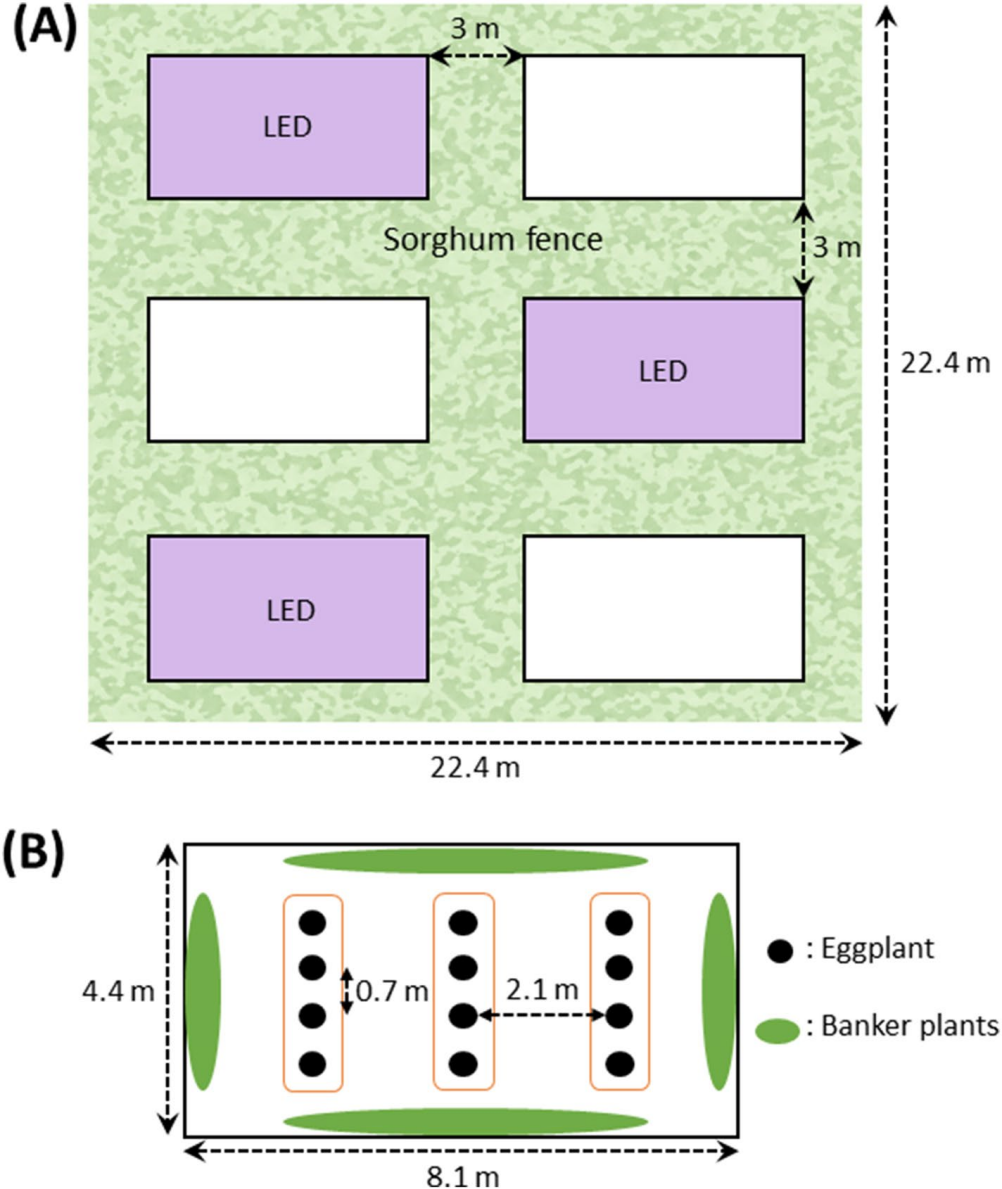
Plot design and locations of experimental plots in the eggplant field. (**A**) The experimental field had six plots which included three illuminated plots and three non-illuminated plots. Sorghum fence approximately 3 m in width were planted to separate each experimental plot to minimize inter-plot interference. (**B**) There were three rows of eggplant per plot. Four eggplant trees were planted in a row. Banker plants were planted surrounding eggplant rows. Space between eggplants was 0.9 m in the row, and one plot consisted of three rows 2.5 m apart.

