# Supplementary material for: Violet LED light enhances the recruitment of a thrip predator in open fields
Source: Sci Rep. 2016 Sep 8;6:32302. doi: 10.1038/srep32302 (PMC5015028; doi:10.1038/srep32302)
Supplement: Supplementary Table 1 [file srep32302-s1.pdf]

Violet LED light enhances the recruitment of a thrip predator in open fields.

Takumi Ogino[1,2,†], Takuya Uehara[1,†], Masahiko Muraji[1], Terumi Yamaguchi[1], Takahisa Ichihashi[3], Takahiro Suzuki[3], Yooichi Kainoh[2] & Masami Shimoda[1,*]

[1] Institute of Agrobiological Sciences, NARO; Ohwashi 1-2, Tsukuba, Ibaraki 305-8634, Japan. [2] Graduate School of Life and Environmental Sciences, University of Tsukuba, Tennodai 1-1-1, Tsukuba, Ibaraki 305-8572, Japan. [3] SHIGRAY Inc., Sumida, Tokyo, Japan. [†]These authors contributed equally to this work.

Correspondence and requests for materials should be addressed to M. S. (E-mail: shimoda1@affrc.go.jp)

Supplementary Table. 1 Schedule of the manipulations performed in the eggplant fields.

Day (2015)		Trial 1 & 2	Trial 3
Apr	30	Eggplants was planted.	
May	8	Sorghum was seeded.	
	13~21	Insectary plants were planted.	
Jun	26	LED was set up and started lighting.	
	29	**Trial 1 was started.**	
Jul	14		Sorghum was seeded.
	22		Eggplants was planted.
	24~28		Insectary plants were planted.
	30	**Trial 1 was ended.**<br>All eggplants were performed the pruning back.	
Aug	4	**Trial 2 was started.**	
	6		Scaevola was planted.
	19		**Trial 3 was started.**
	26		LED was set up and started lighting.
Oct	9	**Trial 2 was ended.**	
	10		**Trial 3 was ended.**

Prior to the experiment, a soil survey was performed and fertilizer was provided as magnesia lime, fused magnesium phosphate, compound fertilizers, and fowl droppings to equalize the status of the soil. Chemical pesticides were not used. The eggplant field was surrounded by a mulberry field.
