# Supplementary material for: Violet LED light enhances the recruitment of a thrip predator in open fields
Source: Sci Rep. 2016 Sep 8;6:32302. doi: 10.1038/srep32302 (PMC5015028; doi:10.1038/srep32302)
Supplement: Supplementary Table 2 [file srep32302-s2.pdf]

Violet LED light enhances the recruitment of a thrip predator in open fields.

Takumi Ogino[1,2,†], Takuya Uehara[1,†], Masahiko Muraji[1], Terumi Yamaguchi[1], Takahisa Ichihashi[3], Takahiro Suzuki[3], Yooichi Kainoh[2] & Masami Shimoda[1,*]

[1] Institute of Agrobiological Sciences, NARO; Ohwashi 1-2, Tsukuba, Ibaraki 305-8634, Japan. [2] Graduate School of Life and Environmental Sciences, University of Tsukuba, Tennodai 1-1-1, Tsukuba, Ibaraki 305-8572, Japan. [3] SHIGRAY Inc., Sumida, Tokyo, Japan. [†]These authors contributed equally to this work.

Correspondence and requests for materials should be addressed to M. S. (E-mail: shimoda1@affrc.go.jp)

Supplementary Table. 2 Numbers of observed other animals on eggplant.

	Trial 1		Trial 2		Trial 3	
	LED	Non-LED	LED	Non-LED	LED	Non-LED
Aphids	54	99	12	16	4	6
Leafhoppers	26	36	11	30	10	10
Frogs	13	14	59	16	17	6

Total numbers of observed aphids, leafhoppers and frogs in each trials were shown this table. Aphids and leafhoppers were captured by stick paper and counted. Frogs were counted by visual observation.
