# Supplementary material for: Violet LED light enhances the recruitment of a thrip predator in open fields
Source: Sci Rep. 2016 Sep 8;6:32302. doi: 10.1038/srep32302 (PMC5015028; doi:10.1038/srep32302)
Supplement: Supplementary Figure 1 [file srep32302-s3.pdf]

Violet LED light enhances the recruitment of a thrip predator in open fields.

Takumi Ogino[1,2,†], Takuya Uehara[1,†], Masahiko Muraji[1], Terumi Yamaguchi[1], Takahisa Ichihashi[3], Takahiro Suzuki[3], Yooichi Kainoh[2] & Masami Shimoda[1,*]

[1] Institute of Agrobiological Sciences, NARO; Ohwashi 1-2, Tsukuba, Ibaraki 305-8634, Japan. [2] Graduate School of Life and Environmental Sciences, University of Tsukuba, Tennodai 1-1-1, Tsukuba, Ibaraki 305-8572, Japan. [3] SHIGRAY Inc., Sumida, Tokyo, Japan. [†]These authors contributed equally to this work.

Correspondence and requests for materials should be addressed to M. S. (E-mail: shimoda1@affrc.go.jp)

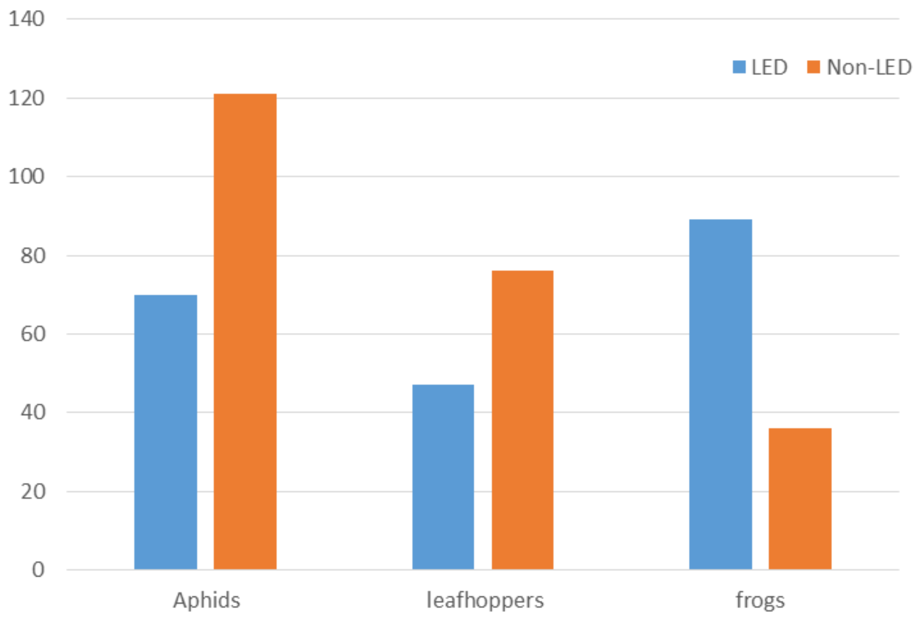

Supplementary Fig. 1
